# Discovery of Innovative Therapies for Rare Immune-Mediated Inflammatory Diseases via Off-Label Prescription of Biologics: *The Case of IL-6 Receptor Blockade in Castleman’s Disease*

**DOI:** 10.3389/fimmu.2015.00625

**Published:** 2015-12-11

**Authors:** Anne Musters, Amira Assaf, Danielle M. Gerlag, Paul P. Tak, Sander W. Tas

**Affiliations:** ^1^Amsterdam Rheumatology and immunology Center; Academic Medical Center, University of Amsterdam, Amsterdam, Netherlands

**Keywords:** giant lymph node hyperplasia, multicentric Castleman’s disease, off-label use, interleukin-6, tocilizumab, biological products, registries

## Abstract

Biologics have revolutionized the field of clinical immunology and proven to be both effective and safe in common immune-mediated inflammatory diseases (IMIDs) such as rheumatoid arthritis, inflammatory bowel diseases, and various hematological disorders. However, in patients with rare, severe IMIDs failing on standard therapies, it is virtually impossible to conduct randomized controlled trials. Therefore, biologics are usually prescribed off-label in these often severely ill patients. Unfortunately, off-label prescription is sometimes hampered in these diseases due to a lack of reimbursement that is often based on a presumed lack of evidence for effectiveness. In the present article, we will discuss that off-label prescription of biologics can be a good way to discover new treatments for rare diseases. This will be illustrated using a case of multicentric Castleman’s disease, an immune-mediated lymphoproliferative disorder, in which off-label tocilizumab (humanized anti-IL-6 receptor blocking antibody) treatment resulted in remarkable clinical improvement. Furthermore, we will give recommendations for monitoring efficacy and safety of biologic treatment in rare IMIDs, including the use of registries. In conclusion, we put forward that innovative treatments for rare IMIDs can be discovered via off-label prescription of biologicals, provided that this is based on rational arguments including knowledge of the pathophysiology of the disease.

## Introduction

In patients with rare, severe immune-mediated inflammatory diseases (IMIDs), biologics are often the last treatment option when standard therapy with classical immunosuppressive drugs fails. However, clear guidelines on biologic prescription in these diseases are often lacking. A major reason is that proper randomized controlled trials (RCTs) are difficult to design due to the low prevalence, heterogeneity of patients, and the severe medical condition of patients. Since RCTs are usually required for registration of therapy for a specific disease, physicians often prescribe biologics off-label in these diseases (1). Off-label prescription can be defined as the prescription of a drug for conditions other than current registered indications. This means that the drug is approved by either FDA or EMA for a certain disease, yet there is no definitive proof for the efficacy of the specific drug for other conditions. The physician’s decision to prescribe off-label in such cases is often based on the pathophysiology of the disease or shared symptoms with other diseases in which the biologic has proven to be effective. This is called rational prescription and may lead to innovative treatment options, especially in rare diseases. We will illustrate this in the present article by discussing the case of Castleman’s disease (CD) and interleukin (IL)-6 receptor blockade, including a case report of a patient who responded very well to this treatment strategy. In addition, we provide an overview of current new developments toward responsible off-label use of biologics in rare, severe, and therapy-refractory IMIDs.

## Castleman’s Disease: Clinical Symptoms and the Role of IL-6

Castleman’s disease is a rare and relatively unknown lymphoproliferative disorder. It is characterized by polyclonal B-cell proliferation, usually associated with autoimmune and connective tissue symptoms, and often goes together with a pre-existing autoimmune disorder, such as rheumatoid arthritis (RA), Sjögren’s syndrome, and systemic lupus erythematodes (SLE) (2).

Castleman’s disease can be divided into unicentric Castleman’s disease (UCD) and multicentric Castleman’s disease (MCD). In UCD, a single lymphoid region is involved, in contrary to the latter in which multiple lymphoid regions are involved (3). UCD usually causes complaints resulting from enlargement of one or more lymph nodes and mostly lacks systemic symptoms (2). Removal of the lymph node cures 90% of the patients without further complications (4). MCD, on the other hand, can cause “B symptoms” and signs such as anorexia, anemia, and low white blood cell counts (3). MCD has been associated with HIV infection, which is similar to MCD observed in non-HIV-infected patients, except for the high prevalence of pulmonary symptoms and strong association with Kaposi’s sarcoma. Interestingly, also HHV-8, a virus causing Kaposi’s sarcoma, is also associated with MCD (5). Both UCD and MCD are characterized by hypersecretion of IL-6, most likely by germinal center B-cells in hyperplastic lymph nodes. Furthermore, a correlation between serum IL-6 levels and clinical symptoms of patients with CD was shown (6). IL-6 regulates T-cell function, acute phase reaction, and terminal B-cell differentiation (2). Overproduction of IL-6 can cause various symptoms, including fever and lymphadenopathy, and has been associated with autoimmune disorders, such as RA and juvenile idiopathic arthritis (JIA), as well as lymphoid malignancies (7). The prognosis for MCD is usually better when diagnosed early. Yet, early diagnosis can be very challenging because of the common manifestation of lymph node enlargement in patients with various autoimmune disorders. Moreover, diagnosing MCD can sometimes be hard, because symptoms are often not very specific. Nonetheless, CD should be suspected when a patient’s primary diagnosed disease, such as another lymphoproliferative disease or autoimmune disease is unusually hard to treat (8).

## Treatment of Castleman’s Disease

Corticosteroids are given as standard therapy in CD, usually resulting in improvement of symptoms, normalization of laboratory parameters, and regression of lymphadenopathy (2, 4). However, relapses are often seen after tapering the dose. Other treatment options are combination chemotherapy and the use of lenalidomide/thalidomine (4). Biologics have also been used in the treatment of CD. Rituximab (chimeric anti-CD20 monoclonal antibody) may be effective, especially in CD patients with pre-existent autoimmune disease (9). Interestingly, long-term remission has also been reported after rituximab administration (2). Clinical improvement after rituximab treatment has been associated with reduced serum levels of pro-inflammatory cytokines, including IL-6 (10). The following case report illustrates the challenges in the treatment of MCD and the potential benefit of rational off-label prescription of biologics in rare IMIDs. In accordance with Dutch laws, the patient gave written informed consent to both the off-label treatment with tocilizumab and the use of his case for this manuscript.

## Case Report

**Figure 1:**
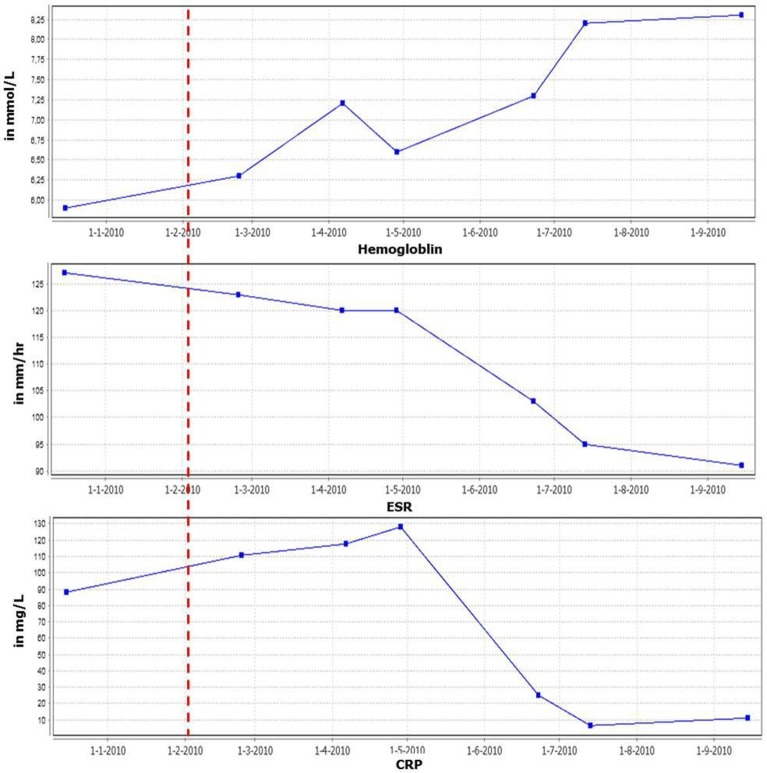
**Inflammatory parameters and hemoglobin levels in time**. Red line indicates start tocilizumab therapy.

A 63-year-old male patient was referred with lymphadenopathy, limb pain, weight loss, and night sweats. Infectious causes and lymphoma were excluded. Eventually, he was diagnosed with MCD, based on clinical manifestations in combination with results of lymph node and bone marrow biopsies demonstrating polyclonal B-cell proliferation. HHV-8 and HIV tests were negative. After failing to respond to high-dose prednisolone treatment, rituximab was administered, dosed in a cycle of 1000 mg intravenously twice every 6 months, which eventually led to a stable condition for 2 years. However, the disease recurred with extensive lymphadenopathy and strongly elevated erythrocyte sedimentation rate (ESR) levels. In addition, he experienced severe weight loss (10 kg), nausea, loss of appetite, increased muscle pain, and extreme fatigue, resulting in disability. Based on the important role of IL-6 in the biology of B-cells, elevated IL-6 levels in patients with CD, and case reports, tocilizumab (anti-IL-6 receptor antibody) treatment was initiated (600 mg biweekly intravenously) combined with methotrexate (7.5 mg weekly). This resulted in a marked decrease in C-reactive protein (CRP) and ESR levels, normalization of hemoglobin (Hb) (Figure 1) and albumin levels and remarkable clinical improvement (weight gain and improvement in overall physical condition). In addition, there was a significant reduction of the lymphadenopathy. Lowering the frequency of tocilizumab administration was not possible as this resulted in immediate flaring of the disease. Subsequent increased dosing induced remission again.

## IL-6 Blockade in Castleman’s Disease

As already mentioned, IL-6 overproduction is associated with the pathogenesis of CD and IL-6 signaling can be blocked by tocilizumab, a humanized monoclonal antibody directed against the human IL-6 receptor. Tocilizumab is used in the treatment of chronic inflammatory diseases in which IL-6 has been implicated in the pathogenesis and exerts its effects by neutralizing the pro-inflammatory effects of IL-6/IL-6 receptor signaling. In addition, tocilizumab treatment may increase the proportion and function of regulatory T cells in the peripheral blood, as has been described in RA (11, 12).

Like most other biologics, the use of tocilizumab is associated with a moderately increased risk of infection. Furthermore, patients sometimes exhibit a transient hypercholesterolemia. Tocilizumab is an effective and safe officially approved treatment for RA and JIA. We describe the successful treatment of a patient with CD with tocilizumab. Over the past few years, several other reports have also described as a good outcome of IL-6 receptor blockade in patients with CD. Nishimoto et al. report an open-label study of 28 patients with MCD. Patients were treated biweekly with 8 mg/kg of tocilizumab for 16 weeks, resulting in improvement of symptoms, reduced acute phase reactants, and decreased need for corticosteroids (13). Interestingly, a recent paper reported persistent improvement of anemia in CD after the administration of tocilizumab. Twelve months after tocilizumab administration, iron-related parameters normalized and symptoms improved in all nine patients (14). This can be explained by the role of IL-6 as inducer of hepcidin, a principal regulator of iron homeostasis (15). Tocilizumab blocks the IL-6 receptor and indirectly downregulates hepcidin. This results in a decrease of iron storage and therefore an increase of serum iron available for Hb synthesis and erythrocyte production, leading to a normalization of the anemia in MCD (2, 14).

As described here, tocilizumab can be very effective in patients with this condition who do not respond to conventional treatment (16). In Japan, these findings have led to the approval and registration of tocilizumab for the treatment of CD (2, 16). However, in many other countries, including the Netherlands and the USA, tocilizumab has not been approved for the treatment of CD and is therefore often not reimbursed.

## Rational Prescription of Biologics and Monitoring Off-Label Use

This case report illustrates that off-label prescription is sometimes the only option left after failure of all other possible treatments and can be very effective. Importantly, off-label prescription of biologics should preferably be “rational,” i.e., prescription of an approved drug proven to be safe and effective in a certain disease for another disease based on shared signs and symptoms or knowledge on the pathophysiology of the disease. The present case is a good example of rational prescription, as the initiation of tocilizumab treatment was mainly based on the important role of IL-6 in the pathophysiology of CD. Other examples of this are the use of anti-TNF biologics in TNF-driven diseases or the prescription of IL-1 receptor antagonists in autoinflammatory diseases that are characterized by derailed IL-1-dependent intracellular processes (1).

Obviously, it is crucial to carefully monitor efficacy and safety of biologics that are prescribed off-label (1). This is not only important for individual patients but also for generating more evidence to eventually support reimbursement of novel off-label treatments for rare IMIDs; especially since healthcare authorities often decide that off-label prescription of these expensive drugs is not reimbursed due to “lack of evidence” for efficacy in rare IMIDs, which may prevent physicians from prescribing biologics in these patients. We therefore advocate improved monitoring and report­ing off-label use of biologics through registries. In several countries, registries have been set up that have provided important information on safety and efficacy in a variety of conditions (17). For example, the RUBRIC registry (acronym for Rational Use of Biologics in Refractory Immune-mediated inflammatory diseases Consortium) is a web-based registry in the Netherlands aiming to identify and monitor all patients suffering from rare therapy-refractory IMIDs who are treated off-label with biologics (18). The information that is generated will be used to develop treatment protocols, which may guide physicians on off-label prescription of biologics and may help payers to make informed decisions about reimbursement.

## Conclusion

Off-label prescription of drugs is both legal and common and may give early access to new valuable treatments for patients, thereby adding to the innovation of clinical practice. This is especially the case in “orphan diseases” such as rare IMIDs, in which it is very difficult to conduct RCTs, but it also holds true for more common diseases as exemplified by infliximab (anti-TNF) treatment for refractory sarcoidosis. However, it is crucially important to consequently monitor efficacy and safety. We therefore advocate the use of registries, which will result in the collection of unbiased data that can be used to develop treatment guidelines for off-label prescription of biologics in individual rare IMIDs. Ultimately, this will lead to a more evidence-based and rational use of biologics in these diseases.

## Author Contributions

AM and AA performed the literature search and wrote the manuscript. AM and AA contributed equally to this paper. DG and PT treated the patient, reviewed, and edited the manuscript. ST wrote and edited the paper.

## Conflict of Interest Statement

The authors declare that the research was conducted in the absence of any commercial or financial relationships that could be construed as a potential conflict of interest.
